# Hepatic focal nodular hyperplasia during follow-up of patients after cyclophosphamide- or oxaliplatin-based chemotherapy: differentiation from liver metastasis

**DOI:** 10.1186/s13244-024-01793-7

**Published:** 2024-08-26

**Authors:** Fan Yang, Wenjing Peng, Shuang Chen, Lijuan Wan, Rui Zhao, Xiangchun Liu, Feng Ye, Hongmei Zhang

**Affiliations:** Department of Radiology, National Cancer Center/National Clinical Research Center for Cancer/Cancer Hospital, Chinese Academy of Medical Sciences and Peking Union Medical College, 100021 Beijing, China

**Keywords:** Focal nodular hyperplasia, Liver metastasis, Cyclophosphamide, Oxaliplatin, Cancer survivors

## Abstract

**Objectives:**

Newly detected hepatic nodules during follow-up of cancer survivors receiving chemotherapy may pose a diagnostic dilemma. We investigated a series of hepatic focal nodular hyperplasia (FNH) diagnosed by either typical MRI features and follow-up or pathology in cancer survivors.

**Methods:**

This retrospective study evaluated 38 patients with tumours who developed new hepatic FNH after cyclophosphamide-based (*n* = 19) and oxaliplatin-based (*n* = 19) chemotherapies. The main tumour types were breast cancer (*n* = 18) and colorectal cancer (*n* = 17). MRI findings, clinical features, and temporal evolution of all target hepatic lesions (*n* = 63) were reported. In addition, the two chemotherapy drug groups were compared.

**Results:**

The median interval between chemotherapy completion and FNH detection was 30.4 months (12.9, 49.4). Six patients underwent biopsy or surgery, while the remaining patients were diagnosed based on typical MRI features and long-term follow-up. Among the patients, 60.5% (23/38) presented with multiple nodules and 63 target lesions were detected. The median size of target lesions was 11.5 mm (8.4, 15.1). The median follow-up time was 32.5 months (21.2, 48.6), and 15 patients experienced changes in their lesions during the follow-up period (11 increased and 4 decreased). The cyclophosphamide-based treatment group had a younger population, a greater proportion of females, and a shorter time to discovery than the oxaliplatin-based chemotherapy group (all *p* ≤ 0.016).

**Conclusions:**

FNH may occur in cancer survivors after cyclophosphamide- or oxaliplatin-based chemotherapy. Considering a patient’s treatment history and typical MRI findings can help avoid misdiagnosis and unnecessary invasive treatment.

**Clinical relevance statement:**

When cancer survivors develop new hepatic nodules during follow-up, clinicians should think of the possibility of focal nodular hyperplasia in addition to liver metastasis, especially if the cancer survivors were previously treated with cyclophosphamide or oxaliplatin.

**Key Points:**

- Cancer survivors, after chemotherapy, can develop hepatic focal nodular hyperplasia.
- Cyclophosphamide and oxaliplatin are two chemotherapeutic agents that predispose to focal nodular hyperplasia development.
- Focal nodular hyperplasia occurs at shorter intervals in patients treated with cyclophosphamide.

**Graphical Abstract:**

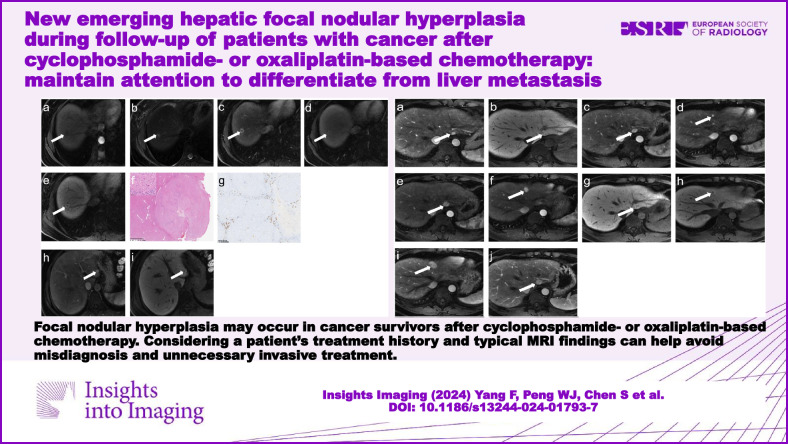

## Introduction

Focal nodular hyperplasia (FNH) is a benign liver tumour originating from the reactive confined proliferation of hepatocytes, with an overall diameter generally smaller than 5 cm [1, 2]. The cause and pathogenesis of FNH remain unclear; however, vascular damage and hepatic sinusoidal endothelial injury may be the potential causes [3]. Meanwhile, it is known that the liver is a common site of distant metastasis for many tumours, such as breast and colorectal cancer. Although liver metastasis is usually the first consideration for newly emerging liver lesions during the follow-up of malignant tumours, it is noteworthy that benign hepatic nodules, especially FNH, may appear in patients with tumours who have received chemotherapy. In clinical practice, hepatic FNH and metastasis require completely different therapeutic strategies, with the latter often being treated aggressively. Unfortunately, misdiagnosis can lead to unnecessary overtreatment, which may further result in psychological burden, high medical expenditure, and low quality of life. Therefore, sufficient understanding and recognition of the hepatic FNH, which can develop in patients with cancer following chemotherapy, is necessary.

Several studies [4–6] have found that hepatic FNH can occur in paediatric cancer survivors after receiving chemotherapy or haematopoietic stem cell transplantation. Chemotherapy-induced liver injury includes portal thrombosis, steatosis, and sinusoidal obstruction syndrome (SOS) [7, 8]. During the liver development period in childhood, chemotherapy may more likely impair normal liver formation, which may lead to hepatic FNH [7, 9]. However, studies on newly emerging liver FNH after chemotherapy in adult patients with malignant tumours are lacking, with only a few case reports. There have been some case reports [10–12] published recently focusing on hepatic FNH after oxaliplatin-based chemotherapy. However, to our knowledge, only one report [13] has described the occurrence of hepatic FNH in a patient with breast cancer after cyclophosphamide-based treatment.

Therefore, in this study, we reviewed medical case data and systematically summarise the MRI characteristics, clinical features, and temporal evolution of FNH in cancer survivors treated with cyclophosphamide or oxaliplatin to contribute to accurate imaging diagnosis and clinical precision therapy.

## Materials and methods

This observational study was approved by the ethics committee of our hospital, and owing to this study’s retrospective nature, the requirement for informed consent was waived. A comprehensive search in Radiology Information System identified 42 patients between January 2010 and December 2022 who met the following criteria: (1) patient who was older than 18 years at the initial diagnosis of primary tumours; (2) patient who completed anti-cancer treatment in our hospital; (3) patient who had a clear diagnosis of FNH for a new hepatic nodule that appears during follow-up; (4) patient who had no evidence of recurrence during the follow-up. Reviewing the medical data from our cancer hospital, we found that the majority of cancer survivors developing FNH were treated with cyclophosphamide or oxaliplatin. To eliminate contingency and spotlight our study, four patients treated with other chemotherapy drugs were excluded. Finally, a total of 38 patients were included in the subsequent analysis.

Clinical information, including age, sex, primary tumour, treatment history, chemotherapy drugs (cycles), the interval between treatment completion and FNH appearance, and follow-up period, were recorded. All patients underwent conventional MRI scans including transverse T1-weighted in-phase and opposed-phase (T1WI), transverse T2-weighted imaging with fat suppression (T2WI/FS), coronal T2WI, diffusion-weighted imaging (DWI, b values = 0 and 800 s/mm^2^), and dynamic contrast-enhanced T1WI at the first and subsequent examinations on 3.0-T MR scanners (Discovery MR 750, GE Healthcare, USA; Siemens, Erlangen, Germany). Gadoxetate disodium (Primovist; Bayer AG) was injected at a rate of 1 mL/s at a dose of 0.025 mmol/kg, and gadodiamide (Magnevist; Bayer AG, Leverkusen, Germany) was injected at a rate of 2 mL/s at a dose of 0.2 mmol/kg. A wash with 20 mL of 0.9% physiological saline flush was used. The transverse images on arterial phase (AP), portal venous phase (PVP), and transitional phase (TP) or delayed phase (DP) were obtained. Transverse hepatobiliary phases (HBP) images were acquired approximately 15 min after gadoxetate disodium injection. Coronal contrast-enhanced T1WI was performed at the final scan. The details of MRI sequences are presented in Table 1.

**Table 1:** MRI scan protocols

		GE Discovery MR750	GE SIGNA Pioneer
T1WI IP-OP (axial)	TR	4.3 ms	4.3 ms
	TE	1.3/2.5 ms	1.3/2.5 ms
	Acquisition matrix	240 × 320	240 × 320
	Slice thickness/space (mm)	4.5/0.5	4.0/0.5
	FOV (mm)	360	420
	Repetition time (ms)	3.9	4.5
	Echo time (ms)	2.2	2.5
T2WI-FS (coronal)	TR	1500 ms	1500 ms
	TE	87 ms	87 ms
	Acquisition matrix	288 × 288	256 × 256
	Slice thickness/space (mm)	4.5/1.0	5.0/1.0
	FOV (mm)	420	420
	Repetition time (ms)	1500	1530
	Echo time (ms)	95	94
T2WI-FS (axial)	TR	Determined by respiratory rate	Determined by respiratory rate
	TE	85 ms	85 ms
	Acquisition matrix	256 × 256	256 × 256
	Slice thickness/space (mm)	4.0/0.5	4.0/0.5
	FOV (mm)	380	380
	Repetition time (ms)	7000	6109
	Echo time (ms)	100	85
DWI (axial)	B values	0/800 s/mm^2^	0/800 s/mm^2^
	TR	Determined by respiratory rate	Determined by respiratory rate
	TE	60 ms	60 ms
	Acquisition matrix	200 × 268	256 × 256
	Slice thickness/space (mm)	5.0/1.0	5.0/1.0
	FOV (mm)	380	380
	Repetition time (ms)	2249	4000
	Echo time (ms)	58	59
CE-T1WI (axial)	TR	3.7 ms	3.7 ms
	TE	1.5 ms	1.5 ms
	Acquisition matrix	288 × 151	240 × 320
	Slice thickness/space (mm)	4.0/0	4.0/0
	FOV (mm)	360	420
	Repetition time (ms)	3.0	3.6
	Echo time (ms)	1.3	1.5

*FOV* field of view, *T2WI* T2-weighted imaging, *T1WI* T1-weighted imaging, *FS* fat suppression, *IP-OP* in-phase and out-of-phase, *DWI* diffusion-weighted imaging, *CE* contrast-enhanced

For each case, the MRI features were recorded, including tumour size, tumour number, image intensity on T1WI, T2WI/FS, DWI, contrast-enhanced AP, PVP, TP or DP, and HBP (if available) images, presence of a central scar, ring hyperintensity on HBP images (if available), and presence of necrosis or cystic components.

FNH was diagnosed through biopsy, surgery, or imaging with long-term follow-up. The imaging characteristics were interpreted by two radiologists (F.Y. and W.J.P., with 4 and 5 years of experience in cancer radiology, respectively). Any disagreements were resolved by a senior radiologist (with > 20 years of experience in abdominal MRI interpretation). Ring hyperintensity on HBP images was defined as a ring of high signal surrounding a central area of relatively low or isointense signal compared to the surrounding normal liver tissues, which is due to the special expression pattern of OATP8 [14]. A central scar was defined as a central T2 hyperintense and delayed enhancement in the TP/DP images compared to the tumour lesions. Hepatic FNH was diagnosed based on representative imaging features and follow-up [15]: iso-to-hypointensity on T1WI, iso-to-hyperintensity on T2WI/FS, homogeneous hyperintensity on AP images, iso-to-hyperintensity on PVP and TP/DP images (absence of washout), and iso-to-hyperintensity on HBP images. In patients with multiple nodules, lesions > 5 mm in diameter were evaluated and recorded [16]. Moreover, the temporal evolution of the size and number of hepatic lesions was recorded.

All statistical analyses were performed using SPSS software version 26.0. Continuous variables were expressed as medians and interquartile ranges, and categorical data were expressed as percentages. For continuous variables, the Kolmogorov–Smirnov test was used to assess whether the variables were normally distributed or not. Then, an independent sample *t*-test or Mann–Whitney *U*-test was used, as appropriate. For categorical variables, the chi-squared test or Fisher’s exact test was used. Statistical significance was set at *p* < 0.05 (two-sided).

## Results

In total, 63 target lesions (diameter > 5 mm) in 38 patients with cancer were included in this study. The clinical information for each patient is presented in Table 2. Twenty-eight women and ten men were included in our study, with a median age of 43.5 years (38.0, 50.5). Cyclophosphamide-based chemotherapy was administered to patients with breast cancer (*n* = 18), and oxaliplatin-based chemotherapy was administered to patients with colorectal cancer (*n* = 17). One patient with non-Hodgkin lymphoma (NHL) was treated with cyclophosphamide, and two patients with gastric cancer received oxaliplatin-based chemotherapy treatment. Six patients underwent surgery or biopsy of hepatic lesions (Supplementary Table).

**Table 2:** Clinical information of cancer survivors with FNH

Patients (No.)	Age	Sex	Primary tumour	History of treatment	Diagnosis of FNH	Interval between treatment complement and FNH diagnosis (months)
1	37	Female	Breast cancer	Adjuvant TCH (6 cycles)	Pathology	18.0
2	37	Female	Breast cancer	Adjuvant TAC (6 cycles)	Imaging and follow-up	33.1
3	38	Female	Breast cancer	Adjuvant EC-T (4-4 cycles)	Pathology	10.5
4	59	Female	Breast cancer	Neoadjuvant EC (6 cycles)	Imaging and follow-up	116.9
5	28	Female	Breast cancer	Adjuvant EC-T (4-4 cycles)	Imaging and follow-up	42.5
6	50	Female	Breast cancer	Adjuvant TC (4 cycles)	Imaging and follow-up	18.6
7	38	Female	Breast cancer	Adjuvant TC (4 cycles)	Imaging and follow-up	18.9
8	38	Female	Breast cancer	Adjuvant TC (4 cycles)	Imaging and follow-up	12.8
9	45	Female	Breast cancer	Adjuvant TCH (6 cycles)	Imaging and follow-up	8.5
10	35	Female	Breast cancer	Adjuvant TC (4 cycles)	Imaging and follow-up	3.4
11	42	Female	Breast cancer	Adjuvant AC (6 cycles)	Imaging and follow-up	0.5
12	42	Female	Breast cancer	Adjuvant AC-TH (4-4 cycles)	Imaging and follow-up	64.9
13	41	Female	Breast cancer	Adjuvant AC-T (4-4 cycles)	Imaging and follow-up	12.9
14	47	Female	Breast cancer	Adjuvant TC (4 cycles)	Imaging and follow-up	6.5
15	35	Female	NHL	CHOP (8 cycles)	Imaging and follow-up	26.4
16	38	Female	Breast cancer	Adjuvant AC-T (4-4 cycles)	Imaging and follow-up	2.4
17	55	Female	Breast cancer	Adjuvant AC (4 cycles)	Imaging and follow-up	43.6
18	39	Female	Breast cancer	Adjuvant AC-T (4-4 cycles)	Imaging and follow-up	5.3
19	40	Female	Breast cancer	Adjuvant EC-T (4-4 cycles)	Pathology	10.6
20	45	Female	Rectal cancer	Neoadjuvant and Adjuvant XELOX (6 + 10 cycles)	Imaging and follow-up	66.8
21	50	Female	Rectal cancer	Adjuvant XELOX (12 cycles)	Imaging and follow-up	31.1
22	31	Female	Colon cancer	Adjuvant XELOX (9 cycles)	Imaging and follow-up	48.7
23	56	Female	Rectal cancer	Adjuvant XELOX (8 cycles)	Imaging and follow-up	61.9
24	63	Male	Gastric cancer	Adjuvant FOLFOX (10 cycles)	Imaging and follow-up	29.7
25	45	Male	Colon cancer	Adjuvant XELOX (8 cycles)	Imaging and follow-up	51.4
26	59	Female	Rectal cancer	Neoadjuvant and Adjuvant XELOX (6 + 6 cycles)	Imaging and follow-up	36.0
27	38	Male	Colon cancer	Adjuvant FOLFOX (10 cycles)	Pathology	16.0
28	61	Female	Colon cancer	Neoadjuvant Xeloda (2 cycles) and Adjuvant XELOX (6 cycles)	Imaging and follow-up	48.4
29	50	Male	Rectal cancer	Adjuvant XELOX (8 cycles)	Imaging and follow-up	17.8
30	52	Male	Rectal cancer	Adjuvant FOLFOX (9 cycles)	Pathology	55.8
31	42	Male	Gastric cancer	Adjuvant XELOX (8 cycles)	Imaging and follow-up	64.3
32	29	Male	Colon cancer	Adjuvant XELOX (8 cycles)	Imaging and follow-up	23.8
33	59	Female	Rectal cancer	Adjuvant XELOX (8 cycles)	Imaging and follow-up	36.0
34	42	Male	Rectal cancer	Adjuvant XELOX (9 cycles)	Imaging and follow-up	25.9
35	47	Male	Colon cancer	Adjuvant FOLFOX (8 cycles)	Pathology	68.6
36	53	Female	Colon cancer	Adjuvant XELOX (8 cycles)	Imaging and follow-up	61.6
37	46	Female	Rectal cancer	Adjuvant XELOX (8 cycles)	Imaging and follow-up	33.6
38	47	Male	Rectal cancer	Adjuvant XELOX (8 cycles)	Imaging and follow-up	31.0

*TCH* Docetaxel, Cyclophosphamide, Herceptin, *TAC* Docetaxel, Adriamycin, Cyclophosphamide, *EC-T* Epirubicin, Cyclophosphamide, Docetaxel, *EC* Epirubicin, Cyclophosphamide, *TC* Docetaxel, Cyclophosphamide, *AC* Adriamycin, Cyclophosphamide, *AC-TH* Adriamycin, Cyclophosphamide, Docetaxel, Herceptin, *NHL* non-Hodgkin lymphoma

The median interval between completion of chemotherapy and FNH appearance was 30.4 months (12.9, 49.4) for all included patients, with 12.9 months (6.5, 33.1) and 36.0 months (29.7, 61.6) for cyclophosphamide-based chemotherapy group and oxaliplatin-based chemotherapy group, respectively. In this study, 55.3% (21/38) of the patients had multiple lesions at the time of first diagnosis, ranging from two to more than five lesions. A comparison between the two groups is presented in Table 3.

**Table 3:** Comparison between the cyclophosphamide-based chemotherapy group and the oxaliplatin-based chemotherapy group

Characteristics	All Patients (*n* = 38)	Cyclophosphamide-based chemotherapy group (*n* = 19)	Oxaliplatin-based chemotherapy group (*n* = 19)	*p* value
Age	43.5 (38.0, 50.5)	39 (37.0, 45.0)	47 (42.0, 56.0)	**0.016**
Tumour type				**< 0.001**
Breast cancer	18	18	0	
Colorectal cancer	17	0	17	
Others^c^	3	1	2	
Sex				**0.001**
Female	28	19	9	
Male	10	0	10	
Number				0.328
Single	17	10	7	
Multiple	21	9	12	
Time interval^a^	30.4 (12.9, 49.4)	12.9 (6.5, 33.1)	36.0 (29.7, 61.6)	**0.002**
Time interval^b^	32.5 (21.2, 48.6)	36.7 (28.4, 48.2)	25.2 (16.8, 51.0)	0.145
Temporal change^d^				0.603
Stable	21	10	11	
Decrease + increase	16 (5 + 11)	9 (3 + 6)	7 (2 + 5)	

Values are presented as median (interquartile); Values in bold are statistically significant

^a^ The interval between the treatment completion and FNH discovery

^b^ The follow-up time after FNH discovery

^c^ Including a patient with NHL and two patients with gastric cancer

^d^ A patient underwent a surgery and had no follow-up

The MRI characteristics of all hepatic lesions are shown in Table 4. The median size of all target lesions was 11.5 mm (8.4, 15.1), with 12.9 mm (9.2, 17.4) and 10.3 mm (8.2, 13.6) for the cyclophosphamide-based chemotherapy group and oxaliplatin-based chemotherapy group, respectively. All lesions were isointense (*n* = 36) or slightly hypointense (*n* = 27) on T1WI and isointense (*n* = 23) or slightly hyperintense (*n* = 40) on T2WI/FS. Thirty-one and thirty-two lesions showed isointense or slightly hyperintense on DWI. All lesions showed obvious and uniform enhancement on the AP images without washout on the PVP and TP/DP images. Among the lesions for which HBP images were available (*n* = 33), 78.8% (26/33) were sustained hyperintense, and 57.6% (19/33) lesions were observed with a ring hyperintense pattern. Nine lesions had a central scar, and none of the target lesions had cystic or necrotic areas. The features of each target lesion are listed in the Supplementary Table.

**Table 4:** MRI characteristics of 63 FNH lesions in 38 patients

Characteristics	All target lesions (*n* = 63)	Cyclophosphamide-based chemotherapy group (*n* = 24)	Oxaliplatin-based chemotherapy group (*n* = 39)	*p* value
Size (mm)	11.5 (8.4, 15.1)	12.9 (9.2, 17.4)	10.3 (8.2, 13.6)	0.144
T1WI				0.238
Slightly hypointense	27	8	19	
Isointense	36	16	20	
T2WI				0.900
Isointense	23	9	14	
Slightly hyperintense	40	15	25	
DWI				0.537
Isointensity	31	13	18	
Slightly hyperintensity	32	11	21	
Hyperintense on AP	63	24	39	NA
Washout on PVP and TP/DP	0	0	0	NA
Central scar				0.262
Yes	9	2	7	
No	54	22	32	
Ring hyperintense on HBP (*n* = 33)	19/33 (57.6%)	3/5 (60.0%)	16/28 (57.1%)	1.000

*AP* arterial phases, *PVP* portal venous phases, *TP* transitional phases, *DP* delayed phases, *HBP* hepatobiliary phases, *NA* not applicable, *DWI* diffusion-weighted imaging

Imaging follow-up was available in 37 patients and no patient with evidence of disease progression during the follow-up period (median, 32.5 months; interquartile, 21.2–48.6 months). We observed that 13.5% (5/37) of patients had decreased FNH size, 10.8% (4/37) had increased FNH size, and 8.1% (3/37) had increased FNH number. Furthermore, four patients (10.8%) had increased FNH numbers accompanied by an increase in size.

**Fig. 1:**
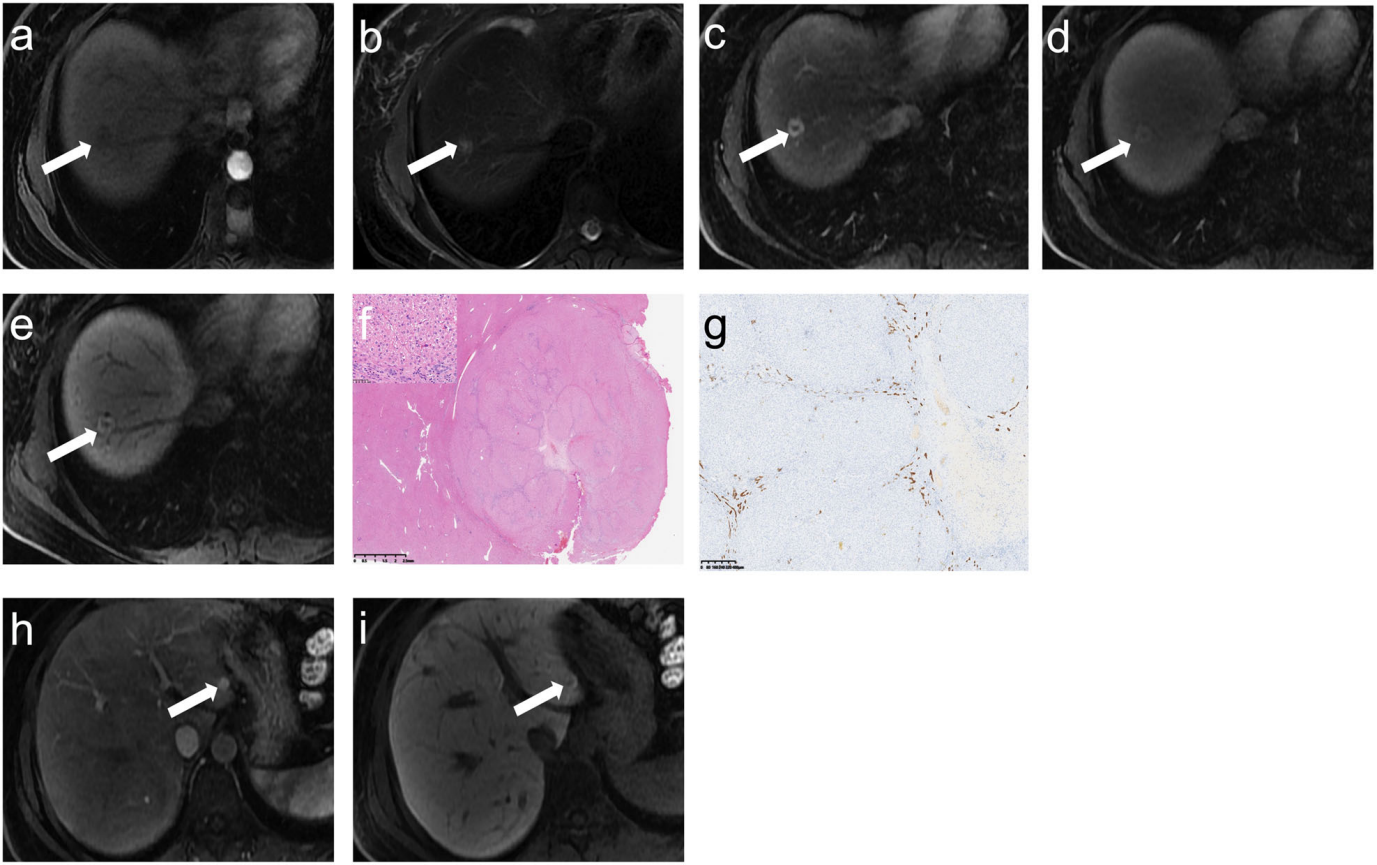
A 37-year-old female with right breast cancer treated with surgery and adjuvant TCH chemotherapy regimen (6 cycles). Gadoxetate disodium enhanced MRI performed 18.0 months after completion of chemotherapy. On segment VII of the liver, lesion 1 (white arrow) showed slightly hypointense on T1WI (**a**), slightly hyperintense on T2WI (**b**), hypointense on AP image (**c**), without washout on DP image (**d**), and ring hyperintense on HBP image (**e**). This lesion was confirmed after laparoscopic resection. On pathological images (**f**), FNH is well-demarcated and has a central radial scar (× 10). The upper left image (× 400) in **f** shows extensive normal hepatocyte aggregates. CD19 staining (**g**, × 400) showed normal bile duct cell morphology within the lesion. Segment IV showed another lesion (white arrow) with hyperintense on AP image (**h**) and HBP image (**i**). AP, arterial phases; DP, delayed phases; HBP, hepatobiliary phases

**Fig. 2:**
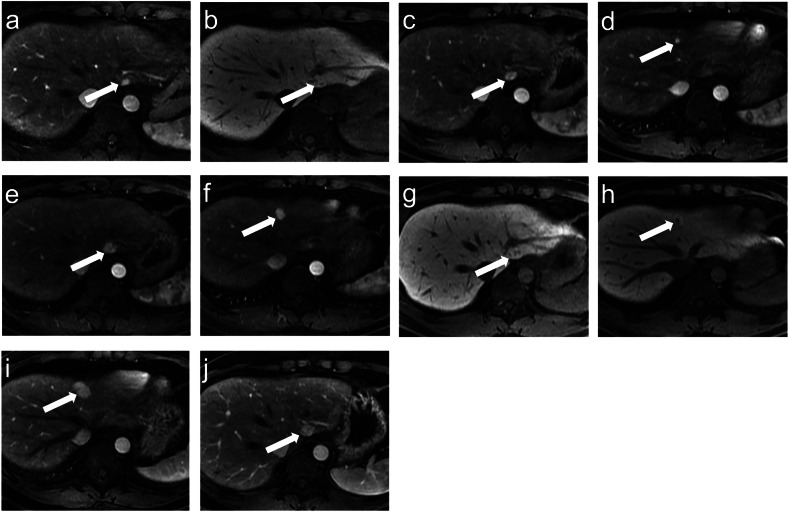
A 29-year-old male with colon cancer treated with surgery and adjuvant XELOX chemotherapy regimen (8 cycles). A hyperintense lesion (lesion 1, white arrow) in AP image (**a**) and HPB image (**b**) was found in segment IV 23.8 months after the completion of treatment. A year later, lesion 1 increased (**c**) in size, and a new hyperintense lesion (lesion 2, **d**, white arrow) was also seen. **e**–**h** A year later, two lesions further increased in size, and both lesions showed hyperintense in AP images and ring hyperintense in HBP images. **i**, **j** After 79.8 months of follow-up, two lesions further increased in size. AP, arterial phases; DP, delayed phases; HBP, hepatobiliary phases

**Fig. 3:**
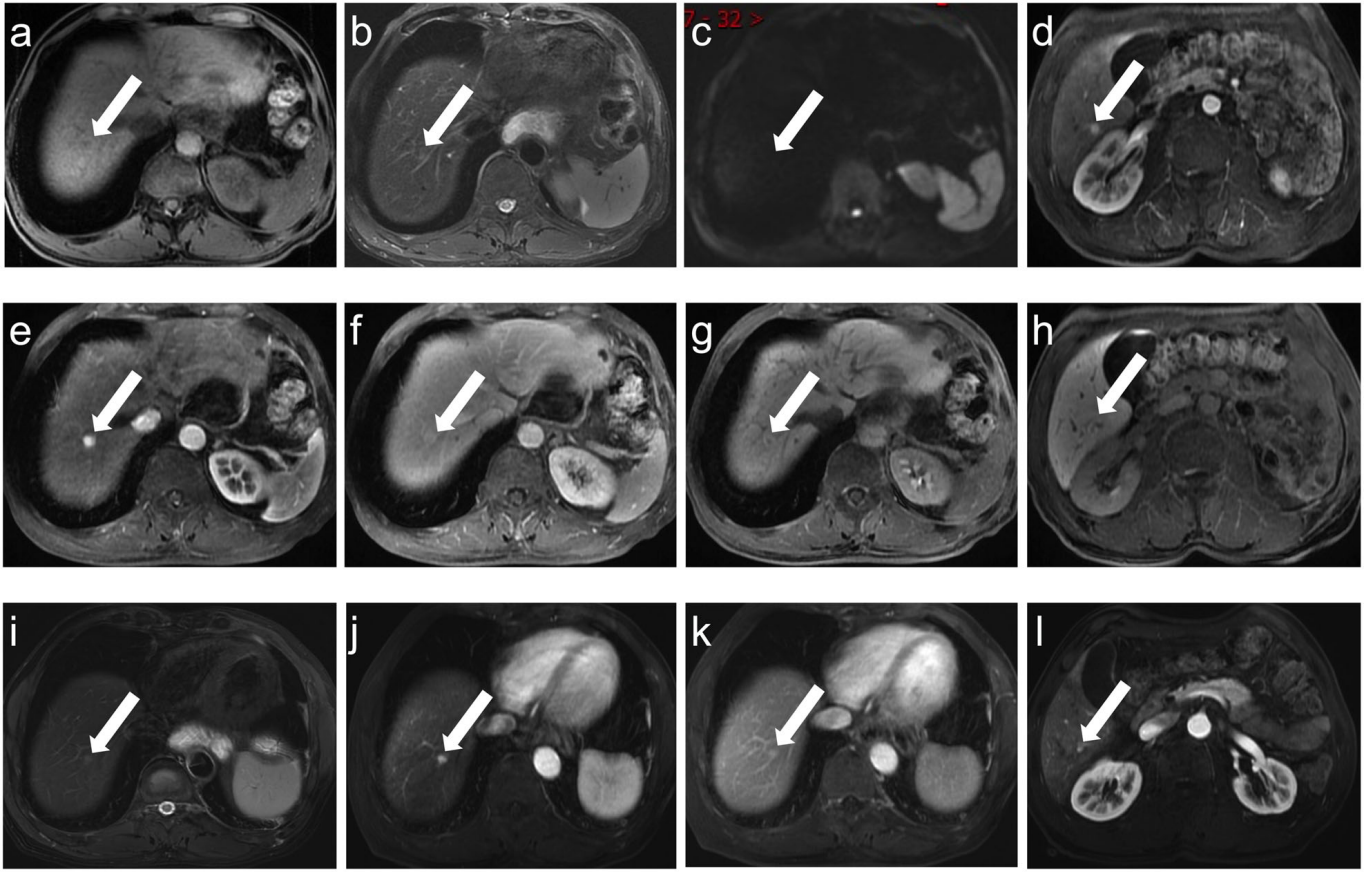
A 63-year-old male with gastric cancer treated with surgery and adjuvant FOLFOX chemotherapy regimen (10 cycles). At 29.7 months after completion of treatment, a nodule (white arrow) showed slightly hypointense on T1WI (**a**), slightly hyperintense on T2WI (**b**), isointense on DWI (**c**), hyperintense on AP image (**e**), without washout on DP image (**f**), and ring hyperintense on HBP image (**g**). Noteworthy, **d** (AP) and **h** (HBP) showed another hepatic FNH. After 49.0 months, T2WI (**i**), AP (**j**, **l**), and DP (**k**) images showed a decrease in the size of both nodules. AP, arterial phases; DP, delayed phases; HBP, hepatobiliary phases

**Fig. 4:**
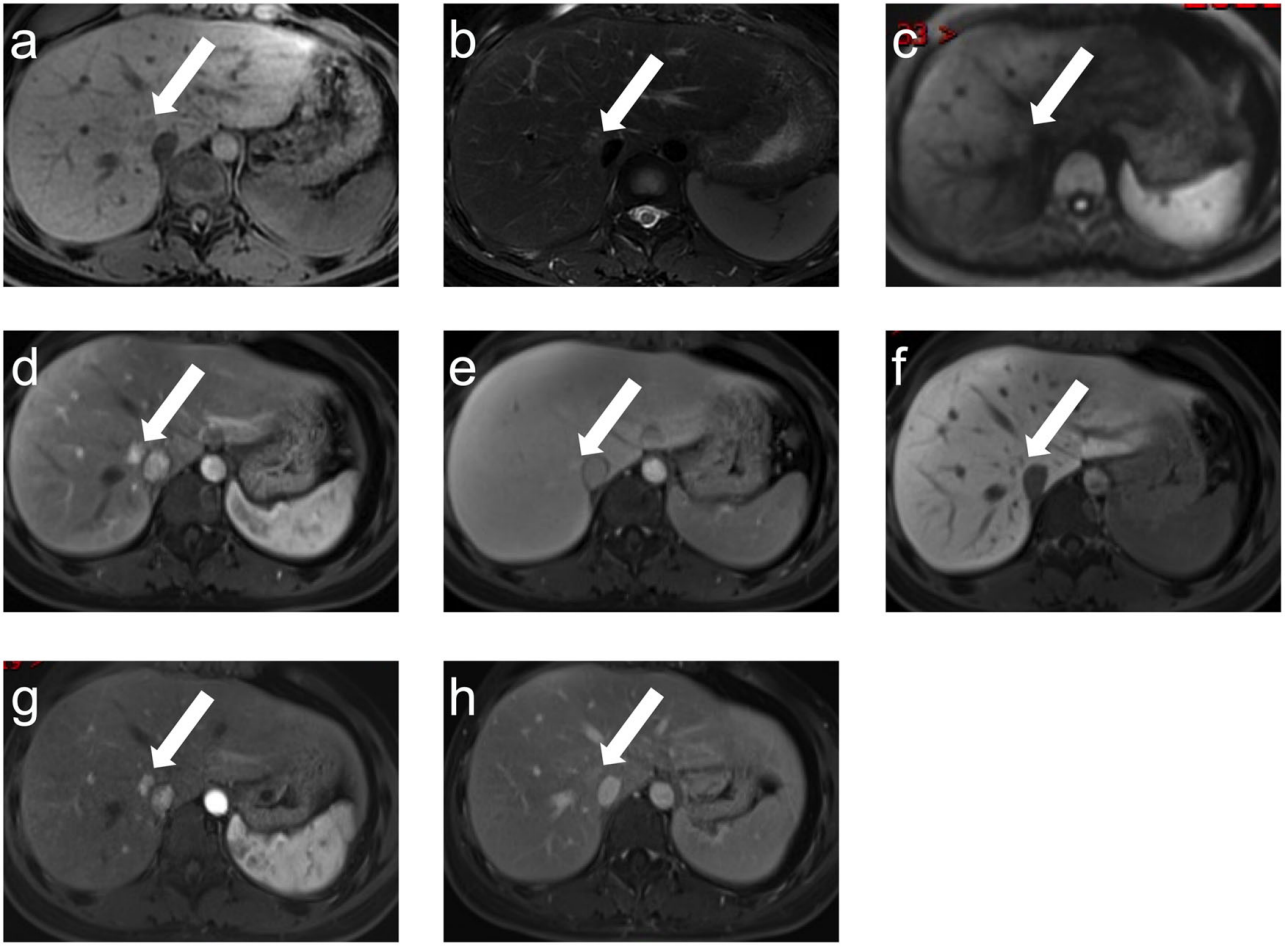
A 28-year-old female with right breast cancer treated with surgery and adjuvant EC-T chemotherapy regimen (6 cycles). A nodule (white arrow) was seen in the hepatic tissue adjacent to the inferior vena cava 42.5 months after the completion of treatment. It showed slightly hypointense on T1WI (**a**), slightly hyperintense on T2WI (**b**), isointense on DWI (**c**), hyperintense on AP image (**d**), without washout on DP image (**e**), and slightly hypointense on HBP image (**f**). After 28.4 months, a decrease in size was seen in AP and DP images (**g**, **h**). AP, arterial phases; DP, delayed phases; HBP, hepatobiliary phases

Comparing two groups of patients using different chemotherapeutic drugs, patients treated with cyclophosphamide were younger (*p* = 0.016), had a greater proportion of females (*p* = 0.001), and had a shorter time from chemotherapy to FNH discovery (*p* = 0.002) than patients treated with oxaliplatin. Representative images are shown in Figs. 1–4.

## Discussion

With the increasing number of cancer survivors, it is crucial to diagnose new hepatic nodules that occur during follow-up accurately. Our study systematically reported 38 patients with 63 hepatic FNH lesions in breast cancer, colorectal cancer, and other scattered types of cancer (NHL and gastric cancer) after chemotherapy. To our knowledge, this is the first relatively large case series to focus on newly emerging hepatic FNH after chemotherapy in adult patients with cancer. This study demonstrated that in addition to paediatric tumours, adult tumours, especially breast and colorectal cancers, could also develop FNH after chemotherapy, particularly after using cyclophosphamide or oxaliplatin, which is very important for radiologists and oncologists to provide accurate diagnosis and treatment decisions.

FNH incidentally discovered on common physical examination usually has typical imaging findings for a relatively easy diagnosis [2]. MRI is an effective diagnostic method with a specificity and sensitivity of 98% and 70%, respectively [17]. Hepatic FNH primarily comprises hepatocytes and resembles the surrounding liver parenchyma on unenhanced MR. The feeding artery and branching vessels in the hepatic FNH accounted for the homogeneous hyperintensity on AP images. Although almost all FNH cases have central scars composed of myxomatous and fibrous elements, they are not always visible on CT and MRI, especially when the nodules are small [18]. The presence of a central scar, which always shows hypointensity on T1WI, hyperintensity on T2WI, and delayed enhancement on contrast-enhanced MR, can improve diagnostic accuracy. In addition, typical uptake patterns using hepatocyte-specific contrast agents on HBP images (iso-hyperintense) are essential to exclude malignancies. Bilreiro et al [19] found that peripheral ring-like hyperintensity on HBP images has excellent specificity (100%) for diagnosing FNH. In our study, approximately two-thirds of the lesions showed this sign on HBP images. The special expression pattern of OATP8 (peripheral rather than central hepatocytes) may be the pathological mechanism underlying the ring hyperintensity pattern in HBP images [20].

Previous studies [1, 15, 16] have found that FNH in cancer survivors has the following characteristics compared to FNH in the general population: multiplicity, small size, and relatively less incidence of the central scar. Approximately 55.3% (21/38) of the patients in our study presented with multiple hepatic FNH lesions at first diagnosis, and two newly presented with multiple lesions during follow-up. The frequency of FNH lesions with a central scar was relatively low (14.3%) in our study, similar to that reported in a previous study (11%) [14]. A plausible explanation is that central scar formation is related to lesion size, and imaging is insensitive to the central scar of small lesions. Kamel et al [21] reported that a central scar occurred in 35% of FNH lesions measuring < 3 cm in size. Strict posttreatment monitoring of patients with tumours allows earlier detection of hepatic FNH, and the FNH lesions in our study were smaller in size (median, 11.5 mm) than those reported in the general population (mean, 43.0 mm) [22].

Previous paediatric FNH studies [4, 14–16] have found that neuroblastoma treated with cyclophosphamide and cisplatin is the most common primary malignancy, accounting for 50–80% of cases. A commonly accepted explanation is that the use of chemotherapy drugs damages the sinusoidal integrity and microvascular function, resulting in the formation of hepatic FNH [1, 2, 23–25]. Particularly, in patients receiving alkylating agent chemotherapy, such as cyclophosphamide, depletion of reduced glutathione in hepatic sinusoidal endothelial cells may be associated with the occurrence of FNH [24–26]. Breast and colorectal cancer were the two main tumour types in our study for newly formed FNH during follow-up, and cyclophosphamide and oxaliplatin were two common chemotherapeutic drugs for these patients. The occurrence of FNH in appendiceal mucinous neoplasms, pancreatic cancer, gastric cancer, and ovarian cancer treated with chemotherapy has been previously reported [4, 11, 27]. However, our study is the first to report a patient with NHL who developed hepatic FNH approximately 2 years after receiving cyclophosphamide treatment. Therefore, the history of chemotherapy, especially the use of cyclophosphamide or oxaliplatin, should be considered a probable risk factor for the occurrence of FNH.

Our study provides a detailed analysis of 38 patients and a comparison of two different treatment groups. The patients in the cyclophosphamide-based chemotherapy group were significantly younger than those in the oxaliplatin-based chemotherapy group. The younger age of onset of breast cancer (approximately 45 years old) than colorectal cancer (approximately 55 years old) may be the main reason. Moreover, the median discovery interval in the cyclophosphamide-based chemotherapy group was 12.9 months, which was significantly shorter than the oxaliplatin-based chemotherapy group (36.0 months). Age and sex differences may account for this result, and the influence of hormone replacement therapy on breast cancer needs to be considered. Previous studies [28, 29] have reported side effects of hormonal medicines in humans, especially in the liver, including thrombosis, steatohepatitis, and hepatic cirrhosis. A higher probability of hepatic vascular injury and recanalization may have accelerated the early onset of FNH in the cyclophosphamide-based chemotherapy group. Oxidative stress, stimulated by hormonal drugs, could also promote FNH growth [30, 31].

None of the patients experienced disease progression (local recurrence or distant metastasis) during the follow-up period. Alteration of hepatic FNH in cancer survivors occurs in approximately 44.4–64.3% of patients [14, 27], and the detection of new hepatic nodules always raises concerns about metastasis, especially when the lesions grow during follow-up. In addition to the typical imaging characteristics, DWI plays a crucial role in differentiating FNH from hepatic metastasis, which is often markedly hyperintense on DWI. In our study, all hepatic nodules were isointense or slightly hyperintense on DWI at the initial diagnosis and subsequent follow-up. Eleven patients in our study experienced an increase in nodule size or number. Despite a history of cancer, a wait-and-see strategy may be the preferred treatment option for asymptomatic patients with typical imaging characteristics [14]. Therefore, knowledge of the possible occurrence of FNH in cancer patients treated with cyclophosphamide or oxaliplatin may effectively prevent aggressive or incorrect treatments. Besides, we noted the lesion shrinkage in four patients, and the same tendency was also seen in previous paediatric studies [6, 14]. We speculated that alternations in hepatic haemodynamics and iron overload in hepatic tissues may be potential reasons for FNH evolution [32].

This study had some limitations. First, most patients did not undergo surgical resection, and pathological information was lacking. Hepatic lesions were diagnosed as FNH based on imaging when they had typical characteristics, and follow-up provided additional information [14, 15]. In our study, three radiologists reviewed a series of imaging data from the remaining patients to ensure the accuracy of our results. Second, the influence of liver background was not included in the statistical analysis. Haemangioma, hepatic hemosiderosis, and iron overloading are associated with the occurrence of multiple FNH lesions and FNH evolution [31–33]. Our study focused on the imaging characteristics of new FNH in patients with cancer, and the influence of the liver background needs to be explored in the future. Finally, hormone replacement therapy in females may be a risk factor for FNH, which perhaps affects the size or number of FNH in imaging follow-up [6]. We did not include this information in our study because various drugs are available as hormonal replacement therapy for breast cancer.

In conclusion, cancer survivors, particularly those treated with cyclophosphamide or oxaliplatin, may present with benign FNH lesions during follow-up. In addition to metastasis, radiologists and clinicians should consider the possibility of FNH in the presence of hepatic nodules on imaging. Typical MRI findings and treatment histories can reduce misdiagnoses and avoid unnecessary invasive treatment.

### Supplementary information

ELECTRONIC SUPPLEMENTARY MATERIAL

## Data Availability

The datasets used and analysed during the current study are available from the corresponding author upon reasonable request.

## Abbreviations

AP: Arterial phase
DP: Delayed phase
DWI: Diffusion-weighted imaging
FNH: Focal nodular hyperplasia
HBP: Hepatobiliary phases
PVP: Portal venous phase
T1WI: T1-weighted imaging
T2WI/FS: T2-weighted imaging with fat suppression
TP: Transitional phase
