# Supplementary material for: Hepatic focal nodular hyperplasia during follow-up of patients after cyclophosphamide- or oxaliplatin-based chemotherapy: differentiation from liver metastasis
Source: Insights Imaging. 2024 Aug 26;15:215. doi: 10.1186/s13244-024-01793-7 (PMC11347512; doi:10.1186/s13244-024-01793-7)
Supplement: Supplementary file 1 — ELECTRONIC SUPPLEMENTARY MATERIAL [file 13244_2024_1793_MOESM1_ESM.pdf]

Hepatic focal nodular hyperplasia during follow-up of patients after cyclophosphamide- or oxaliplatin-based chemotherapy: differentiation from liver metastasis

ELECTRONIC SUPPLEMENTARY MATERIAL

Supplementary Table The characteristics of hepatic FNH lesions in 38 patients

Patients (No.)	Lesion (No.)	Size (mm)	T1WI	T2WI	DWI	AP	PVP	DP	HBP	Central Scar	Follow-up⁺ (months)	Necrosis or Cystic component	Change in lesions⁺
1	1	12.5	s-hypo	s-hyper	s-hyper	hyper	hyper	hyper	NA	No	58.0*	No	stable*
	2	7.8	s-hypo	s-hyper	s-hyper	hyper	hyper	iso	NA	No		No	
2	1	9.1	iso	s-hyper	s-hyper	hyper	iso	iso	NA	No	42.5	No	decrease in size
3	1	19.8	s-hypo	s-hyper	s-hyper	hyper	hyper	hyper	NA	Yes	24.1	No	increase in number
	2	18.3	s-hypo	s-hyper	s-hyper	hyper	hyper	hyper	NA	Yes		No	
4	> 1	7.5	s-hypo	s-hyper	s-hyper	hyper	hyper	hyper	NA	No	53.7	No	increase in size
5	> 1	14.3	iso	iso	s-hyper	hyper	iso	iso	iso	No	28.4	No	decrease in size
6	> 1	10.1	iso	s-	s-	hyper	hyper	hyper	hyper	No	32.5	No	stable

				hyper	hyper								
7	1	19.8	s-hypo	s-hyper	iso	hyper	hyper	hyper	NA	No	44.6	No	stable
8	1	5.4	iso	iso	iso	hyper	hyper	hyper	NA	No	36.2	No	stable
9	1	17.3	iso	iso	iso	hyper	hyper	hyper	NA	No	36.7	No	increase in number
10	> 1	17.4	iso	iso	iso	hyper	hyper	iso	iso	No	26.7	No	stable
11	> 1	11.5	iso	s-hyper	iso	hyper	hyper	hyper	NA	No	76.2	No	increase in number and size
12	1	26.1	iso	iso	iso	hyper	iso	iso	NA	No	31.8	No	stable
13	1	8	s-hypo	s-hyper	s-hyper	hyper	hyper	hyper	NA	No	42.2	No	stable
14	> 1	14.9	s-hypo	iso	s-hyper	hyper	hyper	hyper	NA	No	48.2	No	decrease in size
15	1	15.1	iso	s-hyper	s-hyper	hyper	hyper	hyper	NA	No	85.4	No	increase in size
16	2	13.2	iso	iso	iso	hyper	iso	iso	NA	No	45.1	No	stable
		7.5	iso	iso	iso	hyper	iso	iso	NA	No		No	
17	1	11.2	iso	s-hyper	iso	hyper	iso	iso	NA	No	2.3	No	stable
	2	11.4	iso	s-hyper	iso	hyper	iso	iso	NA	No		No	
18	1	32.1	iso	iso	iso	hyper	iso	iso	NA	No	3.0	No	stable
19	> 2*	13.5	iso	s-hyper	iso	hyper	hyper	hyper	NA	No	35.5	No	increase in size
		9.4	iso	s-hyper	iso	hyper	hyper	hyper	NA	No		No	

20	1	23.4	s-hypo	iso	iso	hyper	hyper	hyper	hyper	Yes	76.5	No	decrease in size
21	> 1	8.9	s-hypo	iso	iso	hyper	hyper	hyper	hyper	No	56.8	No	increase in size
22	4	24.7	iso	iso	s-hyper	hyper	hyper	hyper	NA	No	4.9	No	stable
		16.7	iso	iso	s-hyper	hyper	hyper	hyper	NA	No		No	
		9.3	iso	iso	s-hyper	hyper	hyper	hyper	NA	No		No	
		12	iso	iso	s-hyper	hyper	iso	iso	NA	No		No	
23	> 2	9.6	s-hypo	s-hyper	s-hyper	hyper	hyper	hyper	hyper	Yes	19.6	No	stable
		5.3	s-hypo	iso	s-hyper	hyper	hyper	hyper	hyper	Yes		No	
24	3	10.1	s-hypo	iso	iso	hyper	hyper	hyper	hyper	No	49.0	No	decrease in size
		8.4	iso	s-hyper	s-hyper	hyper	hyper	hyper	hyper	No		No	
		5.6	iso	iso	iso	hyper	hyper	hyper	iso	No		No	
25	> 5	17.6	iso	s-hyper	s-hyper	hyper	hyper	hyper	hyper	No	24.3	No	stable
		13.5	s-hypo	s-hyper	s-hyper	hyper	hyper	hyper	hyper	No		No	
		11.6	iso	iso	iso	hyper	hyper	hyper	hyper	No		No	
		6.1	iso	iso	iso	hyper	hyper	iso	iso	No		No	
		5.2	iso	iso	iso	hyper	hyper	iso	iso	No		No	
26	1	9.5	s-hypo	s-	s-	hyper	hyper	iso	hyper	No	21.2	No	stable

				hyper	hyper								
27	> 1	12.6	s-hypo	s-hyper	iso	hyper	hyper	hyper	NA	No	31.5*	No	increase in number*
28	2	13.1	iso	s-hyper	iso	hyper	hyper	hyper	NA	No	33.3	No	increase in number and size
		13	s-hypo	s-hyper	iso	hyper	hyper	hyper	NA	No		No	
29	3	8.2	iso	s-hyper	iso	hyper	hyper	iso	iso	No	26.1	No	increase in number and size
		6.4	iso	s-hyper	iso	hyper	hyper	iso	hyper	No		No	
		6.2	iso	s-hyper	iso	hyper	hyper	iso	iso	No		No	
30	1	18.4	s-hypo	s-hyper	s-hyper	hyper	hyper	hyper	NA	Yes	NA#	No	NA#
31	3	15.4	iso	s-hyper	s-hyper	hyper	hyper	hyper	hyper	No	59.4	No	stable
		11.6	iso	s-hyper	s-hyper	hyper	hyper	hyper	hyper	No		No	
		10.3	s-hypo	s-hyper	iso	hyper	hyper	hyper	hyper	No		No	
32	> 1	8.9	iso	iso	iso	hyper	hyper	iso	hyper	No	79.8	No	increase in number and size
33	1	10.7	s-hypo	s-hyper	s-hyper	hyper	hyper	hyper	hyper	No	21.2	No	stable
34	1	13.9	s-hypo	s-	s-	hyper	hyper	hyper	hyper	No	15.1	No	stable

				hyper	hyper								
35	1	22.2	iso	iso	s-hyper	hyper	hyper	hyper	hyper	Yes	30.4*	No	stable*
36	3	13.4	s-hypo	s-hyper	s-hyper	hyper	hyper	hyper	NA	Yes	12.3	No	stable
		10.3	s-hypo	s-hyper	s-hyper	hyper	hyper	hyper	NA	No		No	
		8.0	iso	s-hyper	s-hyper	hyper	hyper	hyper	NA	No		No	
37	> 3*	13.6	s-hypo	s-hyper	iso	hyper	hyper	hyper	hyper	Yes	11.8	No	stable
		8.2	iso	s-hyper	iso	hyper	hyper	hyper	hyper	No		No	
		5.7	s-hypo	s-hyper	iso	hyper	hyper	hyper	hyper	No		No	
38	> 2	19.2	s-hypo	s-hyper	s-hyper	hyper	hyper	hyper	hyper	No	17.3	No	stable
		8.9	s-hypo	s-hyper	s-hyper	hyper	hyper	hyper	hyper	No		No	

This table only included the imaging characteristics of hepatic lesions with diameter > 5mm.

$^{+}$, counted on a per-patient basis

$^{\#}$, patient number 30 underwent a surgery and had no follow-up

*, one of the lesions was surgical and the other is in follow-up.

AP, arterial phases; PVP, portal venous phases; DP, delayed phases; HBP, hepatobiliary phases; NA, not applicable; s-hypo, slightly hypointense; iso, isointense; hyper, hyperintense; s-hyper, slightly hyperintense.
